# An Emerging Molecular Understanding and Novel Targeted Treatment Approaches in Pediatric Kidney Diseases

**DOI:** 10.3389/fped.2014.00068

**Published:** 2014-07-01

**Authors:** Max Christoph Liebau

**Affiliations:** ^1^Department of Pediatrics and Center for Molecular Medicine, University Hospital of Cologne, Cologne, Germany; ^2^Nephrology Research Laboratory, Department II of Internal Medicine, University Hospital of Cologne, Cologne, Germany

**Keywords:** podocyte, nephrotic syndrome, aHUS, cilia, polycystic kidney disease, ciliopathies

## Abstract

The evaluation and treatment of the heterogeneous group of kidney diseases poses a challenging field in pediatrics. Many of the pediatric disorders resulting in severe renal affection are exceedingly rare and therapeutic approaches have remained symptomatic for most of these disease entities. The insights obtained from cellular and molecular studies of rare disorders by recent genetic studies have now substantially changed our mechanistic understanding of various important pediatric renal diseases and positive examples of targeted treatment approaches are emerging. Three fields of recent breathtaking developments in pediatric nephrology are the pathophysiology of nephrotic syndrome and proteinuria, the molecular mechanisms underlying atypical hemolytic uremic syndrome, and the genetics and cellular biology of inherited cystic kidney diseases. In all three areas, the combined power of molecular basic science together with deeply characterizing clinical approaches has led to the establishment of novel pathophysiological principles and to the first clinical trials of targeted treatment approaches.

## The Glomerular Filtration Barrier and Proteinuric Disorders: Three Layers for One Task

Glomerular diseases are among the most common reasons for consultation of pediatric nephrologists. Pediatric glomerular disease frequently presents as the symptom complex of nephrotic syndrome characterized by large proteinuria, hypoalbuminemia, edema, and hyperlipidemia (1). While Minimal Change Disease is the classic cause in pediatric nephrotic syndrome, the studies on genetic causes of proteinuric disorders like primary focal and segmental sclerosis (FSGS) have recently improved our understanding of glomerular (patho-)physiology.

**Figure 1:**
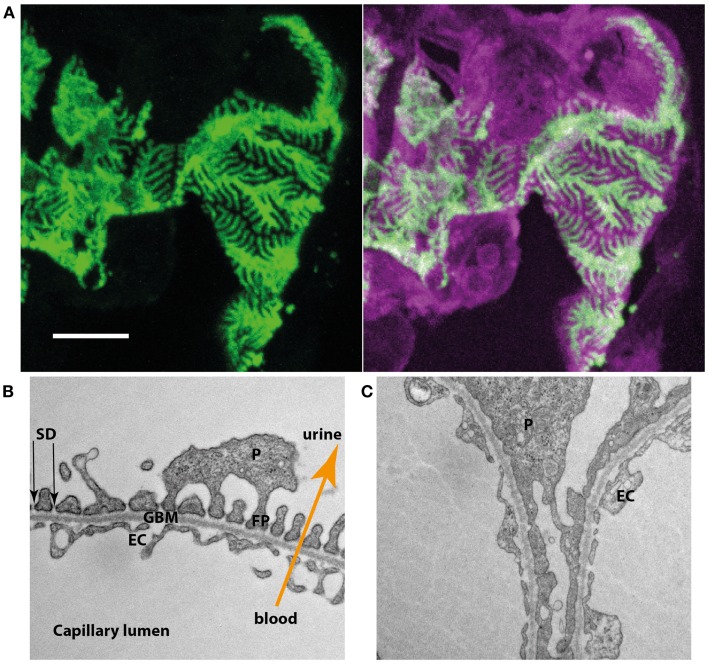
**(A)** Podocyte-specific inducible and mosaic change of expression of membrane-tagged fluorescent proteins proves interdigitation of neighboring podocytes. Induction results in the expression of green fluorescent protein while non-induced cells express a bright red fluorescent protein. Podocytes expressing the different types of fluorescent reporter interdigitate and closely enwrap the glomerular capillaries. **(B)** Electron microscopy of the three layers of the glomerular filtration barrier (EC, endothelial cell; GBM, glomerular basement membrane; P, podocyte; FP, foot process; SD, slit diaphragm). **(C)** In a podocyte-specific proteinuric knockout mouse model secondary foot processes lose their structure and show effacement, a structural change also observed in multiple human proteinuric disorders.

The kidneys constantly filter nearly protein-free primary urine (2). This incredible achievement is accomplished in the glomerulus where the primary urine is derived from the blood perfusing the glomerular capillaries. The wall of these glomerular capillaries forms the glomerular filtration barrier, which consists of three layers. On the blood side a highly specialized fenestrated endothelium is found, which is covering the glomerular basement membrane (GBM). On the outside of the GBM the podocyte, a most remarkable cell type, can be found. Podocytes are characterized by a large cell body with arising primary and secondary foot processes. Secondary foot processes of neighboring podocytes interdigitate and closely enwrap the whole surface of the GBM (Figure 1A). Between the interdigitating foot processes, a slit is formed that is covered by an electron-dense membrane like structure. This so-called slit diaphragm (SD) and the secondary foot processes form the third layer of the glomerular filtration barrier (Figure 1B). In most proteinuric glomerular disorders the secondary foot processes show dramatic structural changes, termed foot process effacement (Figure 1C).

The debate on the question which layer of the filtration barrier would act as “the real filter” was ongoing over decades (3–5). It was therefore of major importance when Karl Tryggvason’s group identified the genetic cause of the rare, but most severe Congenital Nephrotic Syndrome of the Finnish Type. Truncating mutations in *NPHS1*, encoding the SD-protein nephrin, result in this devastating disorder (6, 7). Since the detection of *NPHS1* multiple genes have been identified that are associated with FSGS or familial proteinuria (2, 8, 9). Most of the corresponding gene products either localize to the SD or are crucial for impaired podocyte function thus confirming a central role for podocytes in glomerular disease. Podocyte biology has therefore become a major field of renal basic science. Importantly, it was shown that the SD does not function as a passive glomerular sieve, but that it rather regulates intracellular signaling cascades, e.g., controlling actin polymerization in this structurally highly complex cell type (2, 9). Many of the proteins affected in inherited forms of nephrotic syndrome have been found to form common protein complexes and to functionally cooperate, e.g., in the regulation podocyte cell survival (2, 8, 9).

Still, SD changes are not exclusively responsible for the development of proteinuria. The GBM is affected in genetic proteinuric disorders like Alport’s syndrome or Pierson syndrome (10) and proteinuria precedes detectable podocyte changes in a mouse model of Pierson syndrome (11). Furthermore, alterations in the fenestrated glomerular endothelium can also result in states of proteinuria (12). These fenestrae within the endothelium develop under the influence of vascular endothelial growth factor (VEGF) that is locally generated by podocytes and dysregulation of podocyte-produced VEGF results in proteinuria and endotheliosis (13). Clinical situations resulting in proteinuria due to inhibition of glomerular VEGF function are, e.g., treatment with VEGF antagonists during oncologic therapy or pre-ecclampsia with elevated serum levels of soluble fms-like tyrosine kinase-1 (sFLT-1) that binds and inactivates VEGF (14, 15). The insight into this pathomechanism has recently led to a pilot study on the removal of sFLT-1 in pre-ecclampsia (14).

Given these findings on all three components, the glomerular filtration barrier is nowadays rather seen as a single functional unit than as three independent layers (16, 17). It is the joint action of endothelium, GBM, and podocytes that keeps the filtration barrier working (16, 17).

How do these findings on cellular mechanisms affect our daily clinical work? A very good example is the way we treat steroid-resistant nephrotic syndrome, e.g., in primary FSGS. Primary FSGS results from podocyte injury, is often difficult to treat and frequently progresses to end stage renal disease (ESRD) (18). Currently, a widely accepted treatment approach will escalate immunosuppression in a patient with biopsy-proven FSGS in a primary episode of steroid-resistant nephrotic syndrome. Still, such treatment will be associated with substantial adverse events. Furthermore, podocyte biology backed by recent evidence from clinical observations suggests that immunosuppression will frequently not address, e.g., the genetic cause of primary FSGS and will be ineffective in a number of patients (2, 19). The intensity of immunosuppressive treatment chosen by the pediatric nephrologist will therefore depend on the presence or absence and in some cases potentially on the subtype of a detected mutation (1, 20). As mutations in multiple genes can result in FSGS, age-dependent recommendations for targeted genetic testing have been established (21).

While the decision to include or withhold in immunosuppression in the initial treatment may already be a major reason for genetic testing in these patients, the proof of a mutation in a podocyte-gene has additional important implications for treatment. As chronic kidney disease progresses kidney transplantation may become necessary. For FSGS patients without proof of genetic alterations, it has been suggested that a so-called circulating factor in the blood may be the cause of glomerular damage. The concept of a circulating factor is among other findings based on the observation that around 30% of the patients without genetic alterations show recurrence of FSGS after transplantation (22). Such a recurrence may again be difficult to treat and requires a high level of suspicion as well as rapid therapeutic intervention. In contrast, patients with a genetic alteration affecting SD or podocyte structure will not show recurrence after transplantation and these patients have an excellent prognosis as the intrinsic defect of podocytes will be cured by transplantation. While the idea of a circulating factor has been established for a long time, the factor itself has not been clearly identified. Recent work suggested that soluble uPAR could be a candidate but doubts have risen (23–26). In summary, the recent pathophysiological and clinical insights suggest that we should aim to clearly identify potentially underlying genetic alterations in children with steroid-resistant nephrotic syndrome to individually adapt treatment.

## aHUS, MPGN, and C3GN: “Complementary” Renal Medicine

A second important pediatric renal disease affecting the glomerulus is hemolytic uremic syndrome (HUS). HUS is the most common cause of acute renal failure in childhood (27). It is characterized by microangiopathic hemolytic anemia, thrombocytopenia, and acute renal failure. The vast majority of patients (>90%) show typical HUS due to infection with Shiga-toxin producing *Escherichia coli* (STEC). However, a heterogeneous group of patients with atypical forms of HUS (aHUS) exists, often showing a more severe long-term clinical outcome (27–29). A high percentage of these patients dies or develops ESRD within a year after presentation (30). Recent research has highlighted the role of the alternative pathway of the complement system in the pathogenesis of a large group of aHUS patients. The complement system is a central component of the innate immune system, which is, e.g., involved in the lysis of target cells or bacteria. A crucial step in complement activation is C3 convertase activation and amplification leading to cleavage of C5 and ultimately resulting in the assembly of the membrane attack complex (MAC). Three major pathways activate complement but activation of the so-called alternative pathway is crucial in aHUS (27–29, 31). Mutations in multiple genes encoding proteins associated with the control of the complement cascade C3 convertase complex have been reported in patients with aHUS leading to a hyperactivation of complement on the surface of the endothelium. aHUS may be seen as a paradigm disease for inefficient protection of the endothelium from complement attack (27–29, 31).

Plasma therapy has been the first-line therapy for aHUS for a long time to either replace deficient regulatory complement components or to also remove existing mutant autoantibodies. Still, long-term plasma therapy is associated with substantial morbidity (32–34). Based on the previously described molecular mechanisms, a novel treatment strategy has emerged. Eculizumab is a fully humanized IgG2/IgG4 monoclonal antibody that acts by binding the complement component C5 with high affinity. In this way, Eculizumab inhibits cleavage of C5 and thus the generation of the MAC and has emerged as a powerful treatment approach in aHUS. The effects of Eculizumab in aHUS have recently been described in detail in various excellent reviews (28–30). In summary, efficacy of prevention and treatment of aHUS episodes is greater than with plasma therapy and there are positive results even in case of plasma resistance or dependence. Despite an increased risk of infection especially with *Neisseria meningitidis* under Eculizumab treatment, no severe life-threatening adverse events have been associated with treatment in the few conducted clinical studies (30). It has therefore been suggested that Eculizumab may be considered as a first-line therapy in children with a first episode of aHUS and that Eculizumab may be helpful in patients at risk for post-transplant aHUS recurrence (28, 35). Like for FSGS and proteinuria, the molecular understanding of aHUS has led to the establishment of a novel mechanistic concept and has paved the way for individual decisions on treatment.

In addition to aHUS, membranoproliferative glomerulonephritis (MPGN) and the recently newly classified entity of C3 glomerulonephritis (C3GN) have been linked to activation of the alternative pathway of the complement system (27, 28, 31, 36–42). It has been suggested that complement activation in MPGN and C3GN may take place in the fluid phase rather than on the surface of endothelial cells, thus leading to a phenotype that differs from aHUS. Eculizumab is also considered as a potential therapeutic approach in these patients but more evidence will be needed to establish clear treatment recommendations.

Finally, evidence for complement activation in STEC–HUS exists. First reports on the successful use of Eculizumab in STEC–HUS have been published but data remains ambiguous (27, 43–46). More evidence will be needed, but the insights from rare diseases have helped to shed light into a common pediatric challenge.

## Cystic Kidney Diseases: Small Organelle – Huge Impact

A third common cause of ESRD both in children and adults are polycystic kidney diseases (PKD). The most important forms of pediatric cystic kidney diseases are nephronophthisis (NPH), autosomal recessive polycystic kidney disease (ARPKD), and autosomal dominant polycystic kidney disease (ADPKD) (9, 47–49). ADPKD is the most common genetic cause of ESRD in adults and is among the most common genetic diseases overall, with an incidence of 1:500–1:1000. In children, NPH and ARPKD are currently seen as the most important entities (9, 47–49).

**Figure 2:**
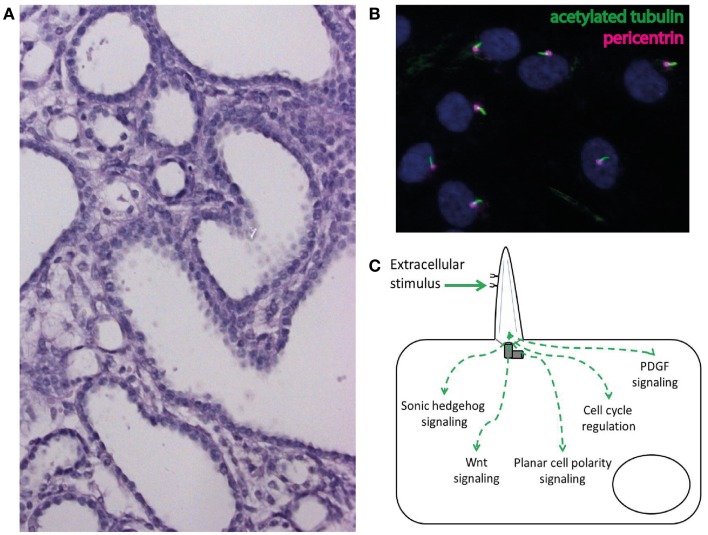
**(A)** Cystic phenotype in a mouse model of ciliary dysfunction. **(B)** Immunofluorescence staining of cilia (acetylated tubulin) and the ciliary base (pericentrin) in human epithelial cells in cell culture. **(C)** Schematic illustration of the involvement of cilia in the regulation of intracellular signaling pathways.

Cysts are fluid-filled epithelium-lined excavations within the kidney (Figure 2A). While a single cyst is frequent and usually benign in adults, even a single cyst should raise suspicion in children and lead to a detailed diagnostic workup looking for a potentially underlying cystic kidney disease that will lead to a progressive loss of renal parenchyma and to ESRD. In addition to the renal findings genetic cystic kidney diseases show typical extrarenal features that may give decisive information to identify the underlying diagnosis (9, 47–49). These extrarenal manifestations are not just a pure coincident but reflect the underlying cellular pathogenesis of genetic cystic kidney diseases. These disorders are nowadays believed to result from dysfunction of a highly – specialized cellular organelle, the primary cilium. Primary cilia are small membrane-bound and microtubule-based protuberances that can be found on nearly all cell types (Figure 2B) (47, 50, 51).

The link between PKDs and cilia was first established when Maureen Barr identified the homolog of the human gene most frequently mutated in ADPKD in the nematode *Caenorhabitis elegans* and could show that the corresponding gene product both localizes to and affects the function of cilia in sensory neurons in the worm (52). The link was strongly supported by the observation that the underlying mutations in a mouse model of PKD affected ciliogenesis and that this mechanism was evolutionarily conserved down to the green algae *Chlamydomonas reinhardtii* (53). In the meantime, nearly all human gene products affected in PKD have been found to localize to cilia (47). The ciliary hypothesis therefore claims that cystic kidney diseases are disorders of the primary cilium, so-called ciliopathies (54). As cilia are present on nearly every human cell type, it becomes obvious, that genetic cystic kidney disease should not just be seen as renal problems but rather are systemic disorders. A ciliary dysfunction in the liver results in congenital hepatic fibrosis in ARPKD and ciliary dysfunction in the retina in subtypes of NPH leads to retinitis pigmentosa. Interestingly, these pathophysiological insights have, e.g., led to the detection of anosmia and defects in peripheral thermo- and mechanosensation in patients with Bardet Biedl syndrome (55, 56).

Over the last 15 years mutations in multiple genes have been identified as the cause of ciliopathies and excellent reviews have recently summarized these findings (47, 48, 50, 51). Mechanistically, cilia have been linked to the regulation of multiple intracellular signaling pathways (Figure 2C) (47, 48, 50, 51). The identification of dysregulated signaling pathways and subsequent promising animal studies have led to the first international randomized controlled clinical trials for the treatment of cilia-associated polycystic renal disorders (49).

Most studies have been performed for ADPKD, including clinical trials on the role of mTOR inhibitors and vasopressin receptor antagonists in ADPKD. A detailed presentation of the underlying pathophysiological considerations is beyond the scope of this review. To most briefly summarize the recent developments, it is crucial to understand the significance of total kidney volume (TKV) for the evaluation of ADPKD treatment approaches. Clinical studies on ADPKD are facing the challenge that renal function in this disease remains stable for a very long time even though substantial structural changes already occur in the kidney. Once renal function starts to decline cystic lesions and fibrosis have massively replaced renal parenchyma and treatment at this late stage will not be able to undo these structural alterations (57). Data from the Consortium on Radiological Investigations in Polycystic kidney disease (CRISP) and the SUISSE study have shown that a rapid increase in TKV, as measured by magnet resonance imaging, is associated with poor outcome in ADPKD (49, 58, 59). It was therefore suggested to monitor TKV as a surrogate parameter for kidney function in clinical studies on ADPKD.

The first major international phase-3 trials for pharmacological treatment of ADPKD focused on mTOR inhibitors (60–62). Data from basic science research including data from multiple mouse models suggested that the mTOR pathway is activated in cyst-lining epithelium in ADPKD (63–65). Treatment with an mTOR inhibitor showed a reduction of kidney growth in these animals. However, the studies on current mTOR inhibitors did not show the hoped-for positive effects in two large European ADPKD studies. While the study by Walz et al. on 433 patients with progressed ADPKD found a significant reduction in kidney volume after 1 year of treatment with everolimus vs. placebo, this effect was not significant any more after 2 years. There was a high dropout rate in the rapamycin group. Surprisingly, the estimated glomerular filtration rate (eGFR) even decreased more rapidly in intervention group (60). Serra et al. found no significant difference in kidney volume or eGFR in 100 less progressed ADPKD patients after 18 months of treatment with sirolimus or placebo, but detected an increase in albuminuria in the sirolimus group (61). The observed results may partly be due to the chosen study designs. Furthermore, it has recently been shown that mTOR signaling in glomerular podocytes requires tight control and proteinuria is a well-known side effect of mTOR inhibition (49, 66–68). A novel generation of modified mTOR inhibitors specifically targeting proliferating cells expressing the folate receptor may become an interesting alternative. Low doses of modified mTOR inhibitors recently showed beneficial effects in murine PKD models (69).

Based on the observation of increased intracellular cAMP in cyst epithelium, increased vasopressin levels in the serum of ADPKD patients and increased levels of V2 receptor expression as well as the knowledge that vasopressin via the V2 receptor increases intracellular cAMP, a second approach suggested that inhibition of the vasopressin-V2R-cAMP axis may reduce cystogenesis an cyst growth (48). Animal data strongly supported hypothesis (70, 71). Both the pharmacological as the genetic interference with vasopressin action showed distinct beneficial effects. The TEMPO 3/4 trial therefore recently studied the effects of the vasopressin antagonist tolvaptan or placebo on 1445 patients with ADPKD and observed a reduction in TKV and loss of renal function after treatment with tolvaptan. However, while there were also less ADPKD-related adverse events, adverse events related to aquaresis occurred as did an increase of liver enzymes. Furthermore, cost-effectiveness of tolvaptan treatment may become an issue (72–74).

Another way to influence intracellular cAMP levels are somatostatin analoga (48). Again, animal data suggests positive effect on PKD and multiple small studies have shown beneficial effects of somatostatin analoga that were also tolerated well (75–78). Recently, the ALADIN trial (a long-acting somatostatin on disease progression in nephropathy due to autosomal dominant polycystic kidney disease), a multicenter, randomized, single-blind, placebo-controlled trial on 79 patients with ADPKD compared octreotide long acting release to placebo (79). There was a significant difference in MRI-measured TKV between control and intervention group after 1 year, but mean TKV increase was only numerically smaller in the octreotide group after 3 years as this effect had lost significance. GFR decline also tended to be less pronounced in the intervention group, but again this effect did not reach significance. Serious adverse events were similarly found in both groups. The data provide evidence for a larger and more powerful follow-up study (79).

The presented developments very clearly illustrate how the novel molecular understanding of cellular events underlying ADPKD has brought up multiple promising treatment approaches for this common cause of ESRD. Furthermore, as cysts start to develop and to grow in childhood, the development of a safe treatment retarding ADPKD progression may also convert this disorder into a major disease for pediatric nephrologists in the near future.

## Conclusion

Over the past years the cellular and molecular studies on rare pediatric renal diseases have resulted in dramatic new pathophysiological insights. Multiple individual signaling cascades and molecular mechanisms crucially involved in the pathogenesis of severe pediatric renal disorders have been identified and novel therapeutic approaches have successfully been established. However, it has also become clear that many of the described signaling pathways are closely interconnected in complex cellular signaling networks in multiple organs and must thus not be seen in isolation. It will now be fundamental to identify and deeply characterize specific subgroups of patients within a single group of disorders. A most detailed clinical phenotyping together with profound molecular studies will expand our functional cellular understanding and may thus open trails for more individualized treatment approaches of severe renal diseases of childhood and adolescence.

## Conflict of Interest Statement

The author declares that the research was conducted in the absence of any commercial or financial relationships that could be construed as a potential conflict of interest.
